# Sex-Specific Expression Patterns of *MYH6* and *MYH7* Gene Transcripts in Large Cohorts of Non-Failing and Failing Human Left Ventricular Tissues

**DOI:** 10.3390/jcdd12110447

**Published:** 2025-11-17

**Authors:** Zdenko Červenák, Ján Somorčík, Yashar Jalali, Žaneta Zajacová, Marian Baldovič, Andrea Gažová, Ján Kyselovič

**Affiliations:** 1 5th Department of Internal Medicine, Faculty of Medicine, Comenius University Bratislava, 813 72 Bratislava, Slovakia; yashar.jalali@fmed.uniba.sk (Y.J.); jan.kyselovic@fmed.uniba.sk (J.K.); 2 Department of Applied Mathematics and Statistics, Faculty of Mathematics, Physics and Informatics, Comenius University Bratislava, 842 48 Bratislava, Slovakia; somorcik@fmph.uniba.sk; 3 Department of Pharmacology and Toxicology, Faculty of Pharmacy, Comenius University Bratislava, 832 32 Bratislava, Slovakia; zajacova76@uniba.sk; 4 Faculty of Natural Sciences, Department of Molecular Biology, Comenius University Bratislava, 842 15 Bratislava, Slovakia; baldovic@ghc.sk; 5 Institute of Pharmacology and Clinical Pharmacology, Faculty of Medicine, Comenius University Bratislava, 813 72 Bratislava, Slovakia; aandreagazova@gmail.com

**Keywords:** myosin gene, DCM, female specific, hypertension, myosin ratio

## Abstract

The transcriptional regulation of *MYH6* and *MYH7* genes has been extensively investigated in healthy versus failing hearts; however, their expression dynamics in healthy human hearts across age and sex, particularly in the context of cardiovascular risk factors such as hypertension, remain poorly characterised. This study aimed to carry out a reanalysis of *MYH6* and *MYH7* transcript levels in a large cohort of non-failing human left ventricular samples, stratified by sex, age, and hypertensive status. Furthermore, we examined how age and sex influence gene expression differences between non-failing and failing hearts, the latter affected by dilated cardiomyopathy (DCM). Normalised expression values for *MYH6* and *MYH7* transcripts from both healthy and failing left ventricles were extracted using the GEO2R online analysis tool from the publicly available RNA-sequencing library GSE141910. This library provides transcriptomic profiles of left ventricular (LV) tissue from both healthy individuals and patients with cardiomyopathies. The Mann–Whitney U test was employed for pairwise comparisons between different groups stratified by sex, age, and hypertensive status. Statistical analysis demonstrates sex-specific differences in *MYH6* and *MYH7* expression in healthy left ventricles, with postmenopausal females (aged > 50 years) with hypertension emerging as a distinct group. Conversely, in end-stage DCM hearts, the expression levels of both myosin genes seemed to be primarily influenced by disease-related pathophysiological mechanisms rather than by sex or age. Comparison between healthy and failing hearts revealed a consistent and significant downregulation of *MYH6* in all comparisons, irrespective of sex or age. On the other hand, *MYH7* expression exhibited greater variability, particularly among males, with age and hypertensive status influencing its expression. The results underscore the importance of considering age, sex, and comorbidities in interpreting cardiac gene expression patterns and highlight potential regulatory divergence in contractile gene expression during cardiac remodelling.

## 1. Introduction

Alterations in gene expression are fundamental drivers of pathological phenotypes. In the human myocardium, such changes often represent adaptive responses to chronic haemodynamic overload and sustained stress, ultimately contributing to the development of cardiac hypertrophy and heart failure [1]. Among the key molecular determinants of cardiac performance are myosin heavy chains (MHC), which function as essential molecular motors driving myocardial contraction. Two principal MHC isoforms are expressed in the human heart. *MYH6* (α-MHC; OMIM 160710) predominantly localises to the atria, typically comprising approximately 80% of the total atrial MHC content, whereas *MYH7* (β-MHC; OMIM 160760) is the predominant isoform in the ventricles, accounting for over 95% of ventricular myosin [2,3,4]. Although these isoforms share a high degree of sequence homology (91% identity within the motor domain), they exhibit distinct biochemical and mechanical properties. Specifically, α-MHC possesses higher ATPase activity, conferring greater contractile velocity, while β-MHC, with a higher tension-time integral per cross-bridge cycle, is more energy-efficient in force generation [5,6,7,8,9]. Consequently, the contractile velocity of the myocardium is closely associated with the relative abundance of each myosin isoform [10]. In the healthy human left ventricle (LV), β-MHC constitutes more than 95% of the total myosin protein. Under pathological conditions such as cardiomyopathy and heart failure, this dominance is further accentuated by a reduction in α-MHC protein levels, often by at least 50% [3]. While isoform shifts at the protein level appear relatively modest, changes at the transcriptional level are considerably more pronounced. In healthy human LVs, *MYH6* transcripts can account for approximately 30–36% of total myosin transcripts, though this proportion varies substantially between individuals [2,4]. In contrast, pathological remodelling is characterised by a pronounced transcriptional shift: *MYH6* mRNA is significantly downregulated to just a few percent of total myosin transcripts, while *MYH7* mRNA increases to nearly 100%, particularly in end-stage heart failure [2,3,4]. These findings suggest that maintaining a physiological *MYH6/MYH7* transcriptional ratio may be essential for preserving normal cardiac function. This notion is further supported by evidence demonstrating that β-blocker therapy in patients with dilated cardiomyopathy (DCM) can partially reverse this maladaptive transcriptional pattern by upregulating *MYH6* and downregulating *MYH7*, an adjustment associated with improved left ventricular ejection fraction [11,12]. Although numerous studies have consistently reported this isoform switch in various cardiomyopathies—including DCM, hypertrophic cardiomyopathy (HCM), ischaemic cardiomyopathy (ICM), and aortic stenosis—comparative data on *MYH6/MYH7* expression ratios in healthy human LVs remain limited. This limitation is largely attributable to the scarcity of normal human heart samples, which often necessitates the inclusion of mixed-sex cohorts without accounting for potential sex-based differences in gene expression. Furthermore, small sample sizes in healthy control groups may also undermine the statistical power of the analyses performed.

In the present study, we investigated *MYH6* and *MYH7* transcript expression in human left ventricles, focusing on sex-based differences in both healthy individuals and those with DCM. Utilising the publicly available RNA-Seq dataset GSE141910, which comprises a large cohort of healthy (n = 161) and DCM (n = 160) samples, we analysed the influence of sex, age, and disease status on myosin gene expression. This study aims to elucidate potential sex-specific differences in the transcriptional regulation of cardiac myosin isoforms under both physiological and pathological conditions and may contribute to a deeper understanding of functional disparities between male and female hearts.

(1) $$\left[a,b\right\}+\left(a;b;c\right)$$

## 2. Materials and Methods

### 2.1. Sample Selection and Data Extraction

The primary dataset used in this study was obtained from the publicly available RNA-sequencing library GSE141910, which provides transcriptomic profiles of left ventricular (LV) tissue from both healthy individuals and patients with cardiomyopathies. The dataset comprises transcriptional profiles from 161 non-failing hearts (75 males and 86 females) representing diverse age groups and ethnic backgrounds, as well as 160 failing hearts diagnosed with dilated cardiomyopathy (DCM) (96 males and 64 females) and 27 samples diagnosed with hypertrophic cardiomyopathy (HCM) (16 males and 11 females) [13].

Normalised expression values for *MYH6* and *MYH7* transcripts from both healthy and failing left ventricles were extracted using the GEO2R online analysis tool [14]. The extracted data are presented in Additional File S1 (Supplementary Materials). Individuals were separated into four categories based on sex and age: females aged ≤ 50 years (younger females), females aged > 50 years (older females), males aged ≤ 50 years (younger males), and males aged > 50 years (older males).

Detailed sample distributions for each subgroup are provided in Additional File S1 (Tables S4–S11).

In the healthy heart cohort, we conducted four primary comparisons of *MYH6* and *MYH7* expression levels: younger females vs. older females, younger males vs. older males, younger females vs. younger males and older females vs. older males.

Additionally, we evaluated the impact of hypertension on *MYH6* and *MYH7* transcript levels within each sex and age category. A relatively large number of samples allowed us to study the effect of hypertension within and between all sex/age combinations. Each sex and age cohort was further divided into two subgroups based on the presence or absence of hypertension, with the exception of younger females with hypertension, where the sample size was insufficient (n = 2). All other subgroups contained a minimum of 10 samples. These hypertension-related subgroups are detailed in Additional File S2. Statistical comparisons were conducted across all subgroups based on age, sex, and hypertension status.

In the DCM cohorts, we performed pairwise comparisons of *MYH6* and *MYH7* expression across all four sex/age groups previously defined (Additional File S1).

Furthermore, we assessed differences in myosin gene expression between healthy and DCM hearts through the following comparisons: all females (healthy) vs. all females (DCM) and all males (healthy) vs. all males (DCM). This analysis was extended to include comparisons between all hypertension-based healthy subgroups and all DCM sex/age subgroups. 

Finally, we examined the *MYH6/MYH7* expression ratio across healthy and DCM hearts. A specific threshold value for this ratio in diseased hearts was established and subsequently used to identify healthy individuals whose *MYH6/MYH7* ratio met or fell below this threshold, with further stratification based on sex, age, and hypertension status.

### 2.2. Statistical Analysis

Given the unequal group sizes and the non-normal distribution of the data, the Mann–Whitney U test was employed for pairwise comparisons. *p*-values were estimated using 10,000 Monte Carlo simulations to ensure accuracy under these distributional constraints. The validity of the Mann–Whitney test for each comparison was verified using the Miller jackknife test to assess the assumption of scale equality. When the Miller jackknife test indicated inequality of scales, the MW results were further validated using the nonparametric Brunner–Munzel test. The Spearman rank correlation coefficient was calculated to evaluate the relationship between *MYH6* and *MYH7* expression levels within all groups (healthy and DCM). For categorical data analyses, Fisher’s exact test and the exact binomial test were applied as appropriate.

All statistical analyses were conducted using GraphPad Prism (GraphPad Software v10, San Diego, CA, USA) and the XLSTAT Excel add-in (Addinsoft, 2025). *p*-value ≤ 0.05 was considered statistically significant. To correct for multiple comparisons, the Hochberg procedure was applied.

## 3. Results

### 3.1. Healthy Hearts

**Figure 1:**
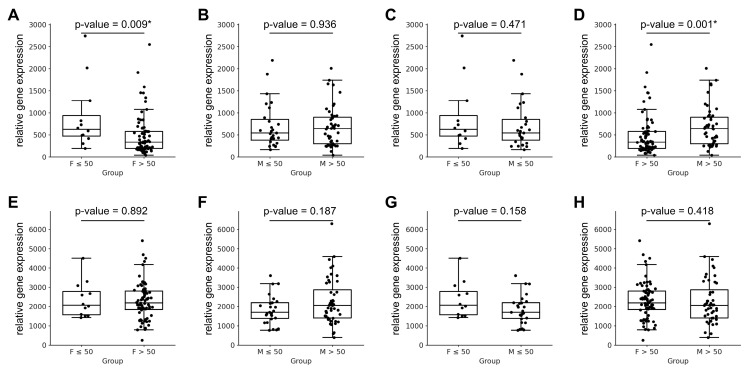
Relative gene expression of *MYH6* and *MYH7* between females and males across age groups. Panels (**A**–**D**) show the relative expression levels of the *MYH6* gene between females and males in the indicated groups, whereas panels (**E**–**H**) depict the corresponding *MYH7* gene expression differences. Data are presented as individual values with the median and interquartile range. Statistical comparisons between females and males were performed using the Mann–Whitney test, and the corresponding *p*-values are shown above each graph. Asterisks (*) indicate *p*-values that remained significant after Hochberg correction for multiple testing. Abbreviations: F ≤ 50—Females aged ≤ 50 years (including 50 y); F > 50—Females aged > 50 years; M ≤ 50—Males aged ≤ 50 years (including 50 y); M > 50—Males aged > 50 years.

The statistical analysis of *MYH6* and *MYH7* transcript expression across all pairwise sex/age comparisons is summarised in Table 1 and Figure 1. The analysis revealed that *MYH6* expression varied significantly between younger and older females (*p* = 0.009) and between older females and older males (*p* = 0.001; Figure 1A–D). In contrast, *MYH7* expression remained stable across all comparisons, with no significant differences observed between any of the groups (Figure 1E–H). This finding suggests that variations in the *MYH6/MYH7* expression ratio within and between groups in the healthy cohort are primarily driven by changes in *MYH6* expression, with little or no contribution from the *MYH7* gene.

An assessment of hypertension prevalence within the four primary groups (analysis of age distribution between groups is present in Additional File S1, Table S1) indicated a significant difference between older and younger females, while no significant differences in hypertension prevalence were found between males of different ages or between sexes within the same age category (Additional File S1, Table S3).

**Figure 2:**
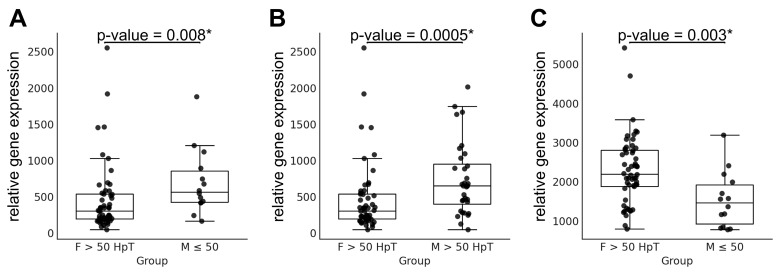
Impact of hypertension on *MYH6* and *MYH7* relative gene expression across sex and age in non-failing human hearts. Panels (**A**,**B**) depict comparisons between hypertensive females aged > 50 years and the corresponding male groups for *MYH6* gene, whereas Panel (**C**) shows the comparison between hypertensive females aged > 50 years and males aged ≤ 50 years for the *MYH7* gene. Data are presented as individual values with median and interquartile range. Statistical differences between females and males were assessed using the Mann–Whitney U test, with *p*-values indicated above each graph. Asterisks (*) denote *p*-values that remained significant after Hochberg correction for multiple testing. Abbreviations are defined in Table 2.

Further pairwise comparisons incorporating hypertension status identified modest differences in *MYH6* expression between older females with hypertension and younger females (*p* = 0.016), as well as between older females and both younger males with hypertension (*p* = 0.020) and older males (*p* = 0.027); Table 2. Importantly, the difference in *MYH6* expression between older females with hypertension and both younger males and older males with hypertension remained statistically significant after correction for multiple testing (Figure 2A,B; Table 2). In contrast, *MYH7* expression was largely stable across the hypertension-stratified comparisons, with only two minor differences detected: younger males without hypertension vs. younger males with hypertension (*p* = 0.022; Table 2) and younger males vs. older males without hypertension (*p* = 0.020; Table 2). Additionally, the comparison between older females with hypertension and younger males without hypertension was unique in that both *MYH6* and *MYH7* were significantly differentially expressed, even after correction for multiple testing (Figure 2A–C). Collectively, these findings indicate that the combined effects of age, sex, and hypertension are most pronounced in the subgroup of older females with hypertension, where the greatest transcriptional differences in *MYH6* (and occasionally *MYH7*) were observed.

Finally, correlation analysis of *MYH6* and *MYH7* transcript expression across all sex, age, and hypertension subgroups (Additional File S3, Table S1) revealed no significant correlations between these two genes in any group tested. The highest coefficient of determination (*R^2^*) was observed in older females with hypertension, although this association did not reach statistical significance and only indicated a non-significant trend.

### 3.2. Failing Hearts

A pairwise analysis, following the same methodology applied to the healthy cohorts (as presented in Table 1), was conducted for the DCM (dilated cardiomyopathy) samples and is summarised in Table 3. This analysis demonstrated that no significant differences in the expression of either *MYH6* or *MYH7* were observed across any of the age/sex comparisons. This indicates a uniform transcriptional profile of these genes within the failing heart cohort, regardless of age or sex.

In contrast to the findings in healthy hearts, correlation analysis in DCM samples revealed a significant positive association between *MYH6* and *MYH7* expression levels across all groups (Additional File S3, Table S2). The coefficients of determination (*R^2^*) were consistently positive and statistically significant, indicating that in failing hearts, the expression levels of these two myosin genes are tightly linked across all age and sex categories. Collectively, these results suggest that in the context of end-stage dilated cardiomyopathy, the transcriptional regulation of *MYH6* and *MYH7* is uniformly affected, independent of factors such as age or sex. Furthermore, the strong positive correlation between *MYH6* and *MYH7* expression in all DCM groups indicates that the pathological remodelling process synchronises the expression of these genes, in contrast to the more variable and independent expression patterns observed in healthy hearts.

### 3.3. Comparison of Healthy and Failing Hearts

**Figure 3:**
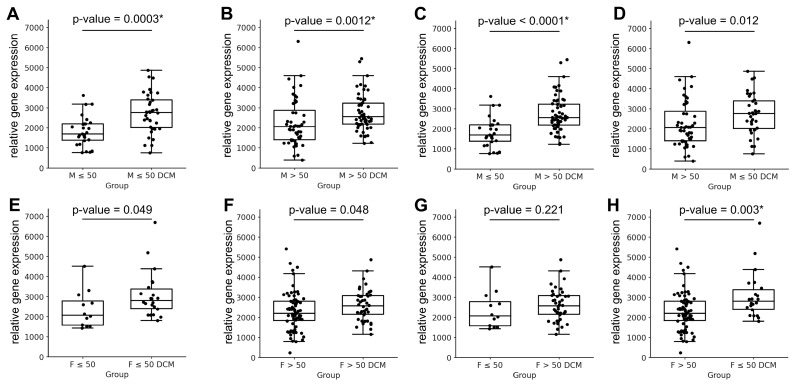
Relative expression of the *MYH7* gene across age- and sex-defined subgroups in control and dilated cardiomyopathy (DCM) samples. Panels (**A**–**D**) illustrate *MYH7* expression comparisons in males, whereas panels (**E**–**H**) display the corresponding comparisons in females. Data are presented as individual values with the median and interquartile range. Statistical differences between groups were assessed using the Mann–Whitney U test, and the resulting *p*-values are shown above each plot. Asterisks (*) denote *p*-values that remained significant after Hochberg correction for multiple comparisons. Abbreviations are consistent with those listed in Table 4.

The comparative analysis of *MYH6* and *MYH7* gene expression between healthy and diseased hearts revealed several key patterns. As summarised in Table 4, *MYH6* expression was consistently downregulated across all comparisons, irrespective of age or sex, confirming its uniform suppression in failing hearts. In contrast, the expression profile of *MYH7* demonstrated more complex, sex-dependent regulation. In all comparisons involving male cohorts, *MYH7* expression was significantly upregulated in failing hearts, consistent with the classic hypertrophic response characterised by *MYH6* downregulation and *MYH7* upregulation (Figure 3A–D). This pattern was observed across all male comparisons, although statistical significance in the comparison between older males and younger males with DCM was achieved at the conventional threshold (*p* ≤ 0.05), but not after correction for multiple testing. For female cohorts, *MYH7* expression changes were more variable. Significant upregulation of *MYH7* was detected only in one comparison—between older healthy and younger diseased females—after correction for multiple testing (Table 4; Figure 3E–H). This suggests that age may modulate *MYH7* expression differently in female hearts, with a less uniform response to disease compared to males.

**Figure 4:**
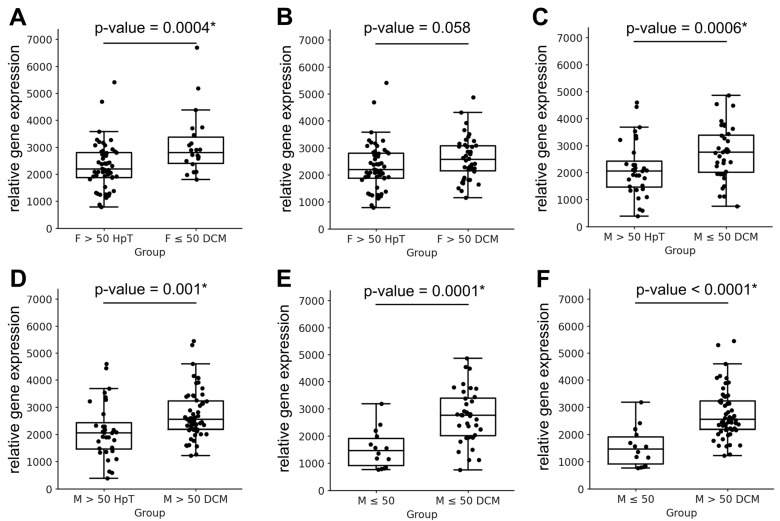
Representative pairwise comparisons of *MYH7* gene expression between control and dilated cardiomyopathy (DCM) groups, stratified by sex, age, and hypertension status. These examples were selected from a broader set of analyses to illustrate characteristic differences across clinical subgroups (Table 5). Data are shown as individual values with the median and interquartile range. Statistical comparisons were performed using the Mann–Whitney U test, with *p*-values displayed above each plot. Asterisks (*) denote *p*-values that remained significant after Hochberg correction for multiple comparisons. Abbreviations correspond to those listed in Table 5.

To further investigate the impact of hypertension and age on myosin gene expression, we compared previously established healthy subgroups, stratified by hypertension status, with DCM samples of both sexes and different age groups. The results of this analysis are presented in Table 5 and Figure 4.

In females, comparisons between older healthy subgroups (with or without hypertension) and older DCM females revealed no significant differences in *MYH7* expression. However, comparisons involving younger DCM females were significant at the *p* ≤ 0.05 level, with statistical significance retained after correction for multiple testing only when the comparison included older healthy females with hypertension (Figure 4A,B).

In males, the expression pattern was distinct. Compared to normotensive older males, the subgroup of older males with hypertension exhibited significantly lower *MYH7* expression relative to failing male hearts, regardless of the age of the diseased group (Figure 4C,D). In contrast, in younger males, only normotensive comparisons showed significant downregulation of *MYH7* after correction for multiple testing (Figure 4E,F). These findings indicate that hypertension is a more influential factor in *MYH7* expression differences among older males, whereas its impact appears less pronounced in younger males.

**Figure 5:**
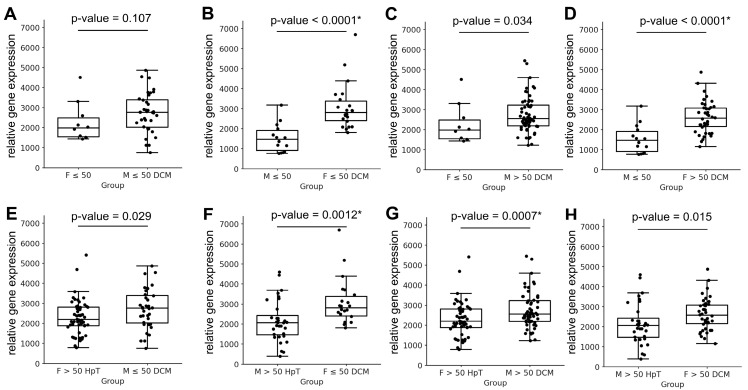
Representative pairwise comparisons of *MYH7* gene expression between male and female subgroups, selected from a larger set of analyses presented in Table 6. The plots illustrate characteristic sex-, age-, and hypertension-associated differences observed in control and dilated cardiomyopathy (DCM) samples. Data are shown as individual values with median and interquartile range. Statistical differences between groups were evaluated using the Mann–Whitney U test; *p*-values are shown above each graph. Asterisks (*) indicate *p*-values that remained significant after Hochberg correction for multiple testing. Abbreviations correspond to those listed in Table 6.

The between-sex comparisons demonstrated that *MYH7* expression was generally associated with the sex of the diseased cohort (Table 6). For example, comparisons between younger healthy females and younger diseased males revealed no significant differences in *MYH7* expression (*p* = 0.107; Figure 5A). However, the inverse comparison—between younger healthy males and younger diseased females—showed significant *MYH7* expression differences (*p* < 0.0001; Figure 5B). Similar sex-dependent trends were observed in most other pairwise comparisons (Figure 5C–H). As not all cross-sex comparisons achieved statistical significance, the overall pattern suggests that future studies on *MYH7* expression and its therapeutic modulation in the human left ventricle should consider sex-specific and potentially age-specific stratification (Table 6).

In contrast to *MYH7*, *MYH6* expression was consistently and significantly downregulated in all comparisons, regardless of age, sex, or hypertension status. Unlike *MYH7*, the direction and magnitude of *MYH6* expression changes did not depend on the sex of the diseased cohort. Thus, our data indicate that the predominant factor influencing *MYH6* downregulation was the presence of cardiac disease itself (Table 6).

### 3.4. Threshold Analysis

A reduced *MYH6/MYH7* gene expression ratio is a well-established molecular hallmark of cardiomyopathy and heart failure. Based on this, we aimed to investigate the distribution of this ratio within the healthy heart samples. For this analysis, we established a threshold of 10% for the *MYH6/MYH7* ratio, reflecting the proportion of *MYH6* transcripts relative to total myosin gene expression. Our analysis demonstrated that over 94% of samples (151/160) in the DCM cohort exhibited an *MYH6/MYH7* ratio at or below this 10% threshold, independent of age or sex. In contrast, within the non-failing (control) group, 39 samples were identified with *MYH6/MYH7* ratios below the established threshold, indicating that based solely on this transcript ratio, these samples are essentially indistinguishable from those in the DCM cohort.

Further statistical evaluation of these 39 control samples revealed a significant over-representation of females compared to males (binomial exact test, *p* = 0.01) and a significantly higher proportion of females over 50 years of age compared to males in the same age group (binomial exact test, *p* = 0.004). The presence of hypertension within this subgroup of females was not significantly different from its expected frequency in the general population of the same age (binomial exact test, *p* = 0.329). Additionally, when comparing the occurrence of hypertension between thresholded and non-thresholded older females in the RNA-Seq dataset, no significant difference was observed (Fisher’s exact test, *p* = 0.788).

In summary, older females with hypertension and under the threshold represented 35% of all females in the higher age group with hypertension, 22.09% of the total number of females in the database and accounted for 49% of all control samples with *MYH6/MYH7* ratios below the threshold. This indicates that older females with hypertension are disproportionately represented within the subset of samples exhibiting a “pathological-like” myosin gene expression profile, despite being classified as non-failing. These findings are further detailed in Additional File S3, Tables S1–S4.

## 4. Discussion

Previous studies examining gene expression in healthy versus failing hearts have typically involved relatively small, age- and sex-matched cohorts. The availability of newly generated RNA sequencing data from a substantially larger cohort has enabled us to reanalyse the expression patterns of *MYH6* and *MYH7* in healthy human left ventricles, considering age, sex, and the presence of hypertension as potential modifiers.

Our analysis revealed significant age-related differences in *MYH6* transcriptional expression among females. However, subsequent inclusion of hypertension status in the analysis indicated that hypertension, rather than age per se, was the primary determinant of these expression differences. Importantly, the observed decline in *MYH6* mRNA levels in females over the age of 50 was not accompanied by a significant upregulation of *MYH7* transcripts. Consequently, changes in the *MYH6/MYH7* ratio in these groups appear to be primarily driven by variations in *MYH6* expression, although the association remains relatively modest. These findings contrast with rodent studies, where an age-dependent increase in *MYH7* expression, at both mRNA and protein levels, has been consistently reported in female rats and mice [15,16]. In our human dataset, no significant changes in *MYH7* expression were observed in females across age groups.

In males, no significant age-dependent alterations were detected in the expression of either myosin gene. When considering hypertension, there was a slight but statistically non-significant change in *MYH7* expression, suggesting limited impact of these variables in males. This contrasts with rodent data, where *MYH7* expression significantly increases with age in male rats and further increases under hypertensive conditions [17].

When comparing sexes, older females with hypertension emerged as a distinct subgroup, particularly regarding *MYH6* gene expression. While *MYH6* expression exhibited minimal variation across female groups, significant differences were observed when comparing older hypertensive females to all male subgroups. *MYH7* expression was comparatively more stable across sexes, though it showed a stronger hypertension- and age-associated variation in older hypertensive females compared to younger males, further reinforcing the unique expression profile of this subgroup.

The differential expression of myosin genes is likely modulated by sex hormones, particularly oestrogens. Animal studies have demonstrated that *MYH7* expression is elevated in young female mice compared to males but decreases to male-equivalent levels following ovariectomy [18]. In rats, ovariectomy reduces *MYH6* protein expression, which can be restored by administration of either testosterone or oestrogen [19]. Our findings suggest that age- and sex-related effects on myosin gene expression in the human left ventricle are primarily driven by *MYH6* transcriptional regulation, with hypertension playing a significant contributory role. Older hypertensive females consistently formed a distinct cluster within the healthy heart cohort.

In contrast, analysis of DCM samples revealed no significant sex- or age-related differences in *MYH6* or *MYH7* expression, suggesting that in the failing heart, transcriptional regulation of these genes is predominantly governed by advanced pathophysiological processes rather than factors like age or sex.

An important observation was that *MYH6* expression was consistently downregulated in all DCM samples compared to healthy controls, irrespective of sex, age, or hypertension status. This underscores the critical role of *MYH6* in maintaining normal left ventricular function. Despite the predominance of *MYH7* protein (~95%) in the human left ventricle, the *MYH6* transcript may play a disproportionately significant role in the heart’s adaptive responses to both physiological and pathological stimuli. Supporting evidence includes (a) that beta-blocker therapy in DCM patients can increase *MYH6* mRNA levels while downregulating *MYH7*, and this shift in the *MYH6/MYH7* ratio is associated with an improvement in left ventricular ejection fraction [11,12]; (b) in failing hearts, not only is *MYH6* mRNA significantly downregulated, but its expression variability is also markedly reduced [2,3], suggesting a “response window” that may be critical for cardiac adaptability.

Comparisons between healthy and failing hearts also demonstrated that *MYH7* expression was less variable among females. In contrast, significant differences in *MYH7* expression were observed in male comparisons, particularly in association with age and hypertension, although these findings were not fully consistent with some prior studies. For example, in patients with aortic stenosis, no significant *MYH7* expression changes were observed in either sex [20], though that study’s cohort included individuals with varying hypertension status and age, which may have confounded results.

Further analysis of non-failing hearts revealed that some samples exhibited *MYH6/MYH7* ratios characteristic of DCM. Specifically, setting a threshold on the *MYH6/MYH7* ratio to 10% (*MYH6* transcript represents 10% or less of the total content of myosin transcripts) identified over 94% of DCM samples as falling below this threshold (151/160), regardless of sex or age. Notably, 39 healthy heart samples also displayed *MYH6/MYH7* ratios below this threshold, with 19 (48%) of females of higher age with hypertension and eight older females without hypertension (21%). These findings indicate that older hypertensive females may represent a distinct subgroup characterised by a reduced *MYH6/MYH7* transcriptional ratio despite the absence of overt cardiac dysfunction. This molecular profile may reflect early or subclinical remodelling of the myocardium. While the dataset used in this study does not include imaging or clinical outcome data to validate this association, the concept is consistent with clinical observations that hypertensive postmenopausal women are at a markedly increased risk for heart failure with preserved ejection fraction (HFpEF), a condition driven by diastolic dysfunction and myocardial stiffening rather than systolic failure [21,22]. The observed transcriptional pattern aligns with previous experimental studies in rodent models [23,24], which demonstrated myosin heavy chain isoform switching even in the absence of hypertrophy, suggesting that these changes may represent an early adaptive or maladaptive response to haemodynamic stress. By extrapolating these findings to the human myocardium, we propose that a reduction in the *MYH6/MYH7* ratio may serve as a preclinical molecular signature of maladaptive remodelling, particularly in older hypertensive women. However, as the discussion now emphasises, it remains unclear whether this pattern represents an early maladaptive process or a transient adaptive response that normalises once the stress stimulus resolves. To resolve this uncertainty, longitudinal studies combining transcriptomic data with imaging and clinical outcomes will be necessary to determine whether such molecular signatures precede the onset of HFpEF or other forms of cardiac dysfunction.

The male group below the threshold (regardless of age and hypertension) was smaller and predominantly included individuals over the age of 50. Given that testosterone levels begin to decline after the age of 40 and that such hormonal changes are associated with increased cardiovascular risk [25], the potential influence of reduced testosterone on myosin gene expression in older males cannot be excluded. However, only 19% of older male samples exhibited a myosin transcript ratio below the 10% threshold, suggesting that additional, yet unidentified factors may contribute to the observed decreased myosin ratio.

## 5. Conclusions

We identified significant sex-based differences in *MYH6* and *MYH7* gene expression within healthy human left ventricles. Our analysis indicates that the downregulation of *MYH6* mRNA is the primary driver of changes in the *MYH6/MYH7* expression ratio when comparing healthy and diseased hearts, underscoring the pivotal role of *MYH6* in the pathophysiology of left ventricular dysfunction. Additionally, our results suggest that the interpretation of gene expression differences between healthy and failing hearts is influenced by the sex of the individual in the diseased cohort, highlighting the necessity of considering sex as a critical variable in cardiac gene expression studies. Furthermore, we identified older females (over 50 years of age) with hypertension as a particularly distinct group that warrants increased attention in the development and testing of new therapeutics aimed at improving the contractile function of the human heart.

We acknowledge several limitations of the present study. First, our analysis is based on a single publicly available RNA-sequencing dataset, and therefore the findings require independent validation using additional cohorts and alternative quantification methods. Second, as no corresponding proteomic data were available, we cannot confirm whether transcript-level changes in *MYH6* and *MYH7* translate directly into alterations at the protein level—a limitation inherent to transcriptomic analyses. Third, because clinical metadata such as detailed medical history or pharmacological treatment were not provided, we cannot exclude that these factors may have influenced gene expression patterns. Fourth, race- or ethnicity-related effects were not analysed due to missing information. Finally, although both thresholds (the age cut-off for group comparisons and the *MYH6/MYH7* ratio threshold) were established based on previously reported physiological and molecular observations, potential modifications of these parameters could yield different results.

## Figures and Tables

**Table 1:** Pairwise comparisons of *MYH6* and *MYH7* gene expression across age and sex groups in non-failing human hearts.

Gene	Comparison	Group 1 (Mean ± SD)	Group 2 (Mean ± SD)	*p*-Value
***MYH6***	F ≤ 50 vs. F > 50	895.33 ± 729.97	486.82 ± 456.97	*0.009* *
	M ≤ 50 vs. M > 50	705.73 ± 498.91	682.69 ± 450.94	0.936
	F ≤ 50 vs. M ≤ 50	895.33 ± 729.97	705.73 ± 498.91	0.471
	F > 50 vs. M > 50	486.82 ± 456.97	682.69 ± 450.94	*0.001* *
***MYH7***	F ≤ 50 vs. F > 50	2358.33 ± 885.88	2295.90 ± 947.47	0.892
	M ≤ 50 vs. M > 50	1834.85 ± 727.39	2263.71 ± 1172.16	0.187
	F ≤ 50 vs. M ≤ 50	2358.33 ± 885.88	1834.85 ± 727.39	0.158
	F > 50 vs. M > 50	2295.90 ± 947.47	2263.71 ± 1172.16	0.418

Statistical analysis was performed using the Mann–Whitney U test; *p* ≤ 0.05 was considered statistically significant; *italicized p*-values indicate statistical significance at the 0.05 level, and asterisks (*) denote *p*-values that remained significant after Hochberg correction for multiple testing. Abbreviations: F ≤ 50—Females aged ≤ 50 years (including 50 y); F > 50—Females aged > 50 years; M ≤ 50—Males aged ≤ 50 years (including 50 y); M > 50—Males aged > 50 years.

**Table 2:** Effect of hypertension on *MYH6* and *MYH7* gene expression across sex and age, in non-failing human hearts.

*MYH6*			
Pairwise Comparisons	Mean ± SD		*p*-Value
F ≤ 50 vs. F > 50	881.30 ± 786.81	569.83 ± 416.04	0.309
F ≤ 50 vs. F > 50 HpT	881.30 ± 786.81	456.07 ± 467.51	*0.016*
F > 50 vs. F > 50 HpT	569.83 ± 416.04	456.07 ± 467.51	0.175
M ≤ 50 vs. M ≤ 50 HpT	699.24 ± 452.72	712.73 ± 581.31	0.822
M ≤ 50 vs. M > 50	699.24 ± 452.72	561.33 ± 354.90	0.395
M ≤ 50 vs. M > 50 HpT	699.24 ± 452.72	734.47 ± 497.21	0.870
M ≤ 50 HpT vs. M >50	712.73 ± 581.31	561.33 ± 354.90	0.790
M ≤ 50 HpT vs. M > 50 HpT	712.73 ± 581.31	734.47 ± 497.21	0.632
M > 50 vs. M > 50 HpT	561.33 ± 354.90	734.47 ± 497.21	0.340
F ≤ 50 vs. M ≤ 50	881.30 ± 786.81	699.24 ± 452.72	0.841
F ≤ 50 vs. M ≤ 50 HpT	881.30 ± 786.81	712.73 ± 581.31	0.738
F ≤ 50 vs. M > 50	881.30 ± 786.81	561.33 ± 354.90	0.397
F ≤ 50 vs. M > 50 HpT	881.30 ± 786.81	734.47 ± 497.21	0.965
F > 50 vs. M > 50	569.83 ± 416.04	561.33 ± 354.90	0.705
F > 50 vs. M > 50 HpT	569.83 ± 416.04	734.47 ± 497.21	0.187
F > 50 vs. M ≤ 50	569.83 ± 416.04	699.24 ± 452.72	0.306
F > 50 vs. M ≤ 50 HpT	569.83 ± 416.04	712.73 ± 581.31	0.478
F > 50 HpT vs. M ≤ 50	456.07 ± 467.51	699.24 ± 452.72	*0.008* *
F > 50 HpT vs. M ≤ 50 HpT	456.07 ± 467.51	712.73 ± 581.31	*0.02*
F > 50 HpT vs. M > 50	456.07 ± 467.51	561.33 ± 354.90	*0.027*
F > 50 HpT vs. M > 50 HpT	456.07 ± 467.51	734.47 ± 497.21	*0.0005* *
***MYH7***			
Pairwise comparisons	Mean ± SD		*p*-value
F ≤ 50 vs. F > 50	2253.20 ± 931.04	2281.46 ± 1141.35	0.914
F ≤ 50 vs. F > 50 HpT	2253.20 ± 931.04	2301.24 ± 864.64	0.669
F > 50 vs. F > 50 HpT	2281.46 ± 1141.35	2301.24 ± 864.64	0.780
M ≤ 50 vs. M ≤ 50 HpT	1535.29 ± 686.24	2157.47 ± 625.31	*0.022*
M ≤ 50 vs. M > 50	1535.29 ± 686.24	2590.00 ± 1408.74	*0.020*
M ≤ 50 vs. M > 50 HpT	1535.29 ± 686.24	2142.65 ± 1016.65	0.057
M ≤ 50 HpT vs. M >50	2157.47 ± 625.31	2590.00 ± 1408.74	0.811
M ≤ 50 HpT vs. M > 50 HpT	2157.47 ± 625.31	2142.65 ± 1016.65	0.795
M > 50 vs. M > 50 HpT	2590.00 ± 1408.74	2142.65 ± 1016.65	0.505
F ≤ 50 vs. M ≤ 50	2253.20 ± 931.04	1535.29 ± 686.24	0.056
F ≤ 50 vs. M ≤ 50 HpT	2253.20 ± 931.04	2157.47 ± 625.31	0.693
F ≤ 50 vs. M > 50	2253.20 ± 931.04	2590.00 ± 1408.74	0.849
F ≤ 50 vs. M > 50 HpT	2253.20 ± 931.04	2142.65 ± 1016.65	0.827
F > 50 vs. M > 50	2281.46 ± 1141.35	2590.00 ± 1408.74	0.780
F > 50 vs. M > 50 HpT	2281.46 ± 1141.35	2142.65 ± 1016.65	0.634
F > 50 vs. M ≤ 50	2281.46 ± 1141.35	1535.29 ± 686.24	*0.039*
F > 50 vs. M ≤ 50 HpT	2281.46 ± 1141.35	2157.47 ± 625.31	0.758
F > 50 HpT vs. M ≤ 50	2301.24 ± 864.64	1535.29 ± 686.24	0.003 *
F > 50 HpT vs. M ≤ 50 HpT	2301.24 ± 864.64	2157.47 ± 625.31	0.557
F > 50 HpT vs. M > 50	2301.24 ± 864.64	2590.00 ± 1408.74	0.914
F > 50 HpT vs. M > 50 HpT	2301.24 ± 864.64	2142.65 ± 1016.65	0.296

Statistical analysis was performed using the Mann–Whitney U test; *p* ≤ 0.05 was considered statistically significant; *italicized p*-values indicate statistical significance at the 0.05 level, and asterisks (*) denote *p*-values that remained significant after Hochberg correction for multiple testing. Abbreviations: F ≤ 50—Females aged ≤ 50 years (including 50 y); F > 50—Females aged > 50 years; M ≤ 50—Males aged ≤ 50 years (including 50 y); M > 50—Males aged > 50 years; HpT—hypertension.

**Table 3:** Pairwise comparisons of *MYH6* and *MYH7* gene expression in age/sex subgroups in dilated cardiomyopathy.

Gene	Comparison	Group 1 (Mean ± SD)	Group 2 (Mean ± SD)	*p*-Value
***MYH6***	F ≤ 50 vs. F > 50	202.15 ± 127.33	160.99 ± 91.52	0.183
	M ≤ 50 vs. M > 50	156.77 ± 89.89	190.19 ± 127.15	0.269
	F ≤ 50 vs. M ≤ 50	202.15 ± 127.33	156.77 ± 89.89	0.175
	F > 50 vs. M > 50	160.99 ± 91.52	190.19 ± 127.15	0.334
***MYH7***	F ≤ 50 vs. F > 50	3076.46 ± 120.87	2613.48 ± 784.86	0.152
	M ≤ 50 vs. M > 50	2736.27 ± 972.66	2743.73 ± 883.05	0.782
	F ≤ 50 vs. M ≤ 50	3076.46 ± 120.87	2736.27 ± 972.66	0.434
	F > 50 vs. M > 50	2613.48 ± 784.86	2743.73 ± 883.05	0.599

Statistical analysis was performed using the Mann–Whitney U test; *p* ≤ 0.05 was considered statistically significant, and Hochberg correction for multiple testing applied. Abbreviations: F ≤ 50—Females aged ≤ 50 years (including 50 y); F > 50—Females aged > 50 years; M ≤ 50—Males aged ≤ 50 years (including 50 y); M > 50—Males aged > 50 years.

**Table 4:** Pairwise comparisons of *MYH6* and *MYH7* gene expression between control and dilated cardiomyopathy (DCM) groups across age and sex subgroups.

*MYH6*			
Pairwise Comparisons	Mean ± SD		*p*-Value
F ≤ 50 vs. F ≤ 50 DCM	895.33 ± 62.42	202.15 ± 30.32	<0.0001 *
F > 50 vs. F > 50 DCM	486.82 ± 60.09	160.99 ± 92.63	<0.0001 *
F ≤ 50 vs. F > 50 DCM	895.33 ± 62.42	160.99 ± 92.63	<0.0001 *
F > 50 vs. F ≤ 50 DCM	486.82 ± 60.09	202.15 ± 30.32	<0.0001 *
M ≤ 50 vs. M ≤ 50 DCM	705.73 ± 508.42	156.77 ± 91.17	<0.0001 *
M > 50 vs. M > 50 DCM	682.69 ± 455.71	190.19 ± 28.23	<0.0001 *
M ≤ 50 vs. M > 50 DCM	705.73 ± 508.42	190.19 ± 28.23	<0.0001 *
M > 50 vs. M ≤ 50 DCM	682.69 ± 455.71	156.77 ± 91.17	<0.0001 *
***MYH7***			
Pairwise comparisons	Mean ± SD		*p*-value
F ≤ 50 vs. F ≤ 50 DCM	2358.33 ± 25.27	3076.46 ± 147.24	*0.049*
F > 50 vs. F > 50 DCM	2295.90 ± 53.94	2613.48 ± 794.37	*0.048*
F ≤ 50 vs. F > 50 DCM	2358.33 ± 25.27	2613.48 ± 794.37	0.221
F > 50 vs. F ≤ 50 DCM	2295.90 ± 53.94	3076.46 ± 147.24	0.003 *
M ≤ 50 vs. M ≤ 50 DCM	1834.85 ± 41.24	2736.27 ± 986.46	0.0003 *
M > 50 vs. M > 50 DCM	2263.71 ± 184.57	2743.74 ± 890.50	0.0012 *
M ≤ 50 vs. M > 50 DCM	1834.85 ± 741.24	2743.74 ± 890.50	<0.0001 *
M > 50 vs. M ≤ 50 DCM	2263.71 ± 184.57	2736.27 ± 986.46	*0.012*

Statistical analysis was performed using the Mann–Whitney U test; *p* ≤ 0.05 was considered statistically significant; *italicized p*-values indicate statistical significance at the 0.05 level, and asterisks (*) denote *p*-values that remained significant after Hochberg correction for multiple testing. Abbreviations: F ≤ 50—Females aged ≤ 50 years (including age 50 y); F > 50—Females aged > 50 years; F ≤ 50 DCM—Females aged ≤ 50 years with dilated cardiomyopathy; F > 50 DCM—Females aged > 50 years with dilated cardiomyopathy; M ≤ 50—Males aged ≤ 50 years (including age 50 y); M > 50—Males aged > 50 years; M ≤ 50 DCM—Males aged ≤ 50 years with dilated cardiomyopathy; M > 50 DCM—Males aged > 50 years with dilated cardiomyopathy.

**Table 5:** Sex-based pairwise comparisons of *MYH6* and *MYH7* gene expression between different age/hypertension control groups, and dilated cardiomyopathy (DCM) groups.

*MYH6*			
Pairwise Comparisons	Mean ± SD		*p*-Value
F ≤ 50 vs. F ≤ 50 DCM	881.3 ± 29.37	202.15 ± 30.32	*<0.0001* *
F ≤ 50 vs. F > 50 DCM	881.3 ± 29.37	160.99 ± 92.63	<0.0001 *
F > 50 vs. F ≤ 50 DCM	569.83 ± 26.84	202.15 ± 130.32	<0.0001 *
F > 50 vs. F > 50 DCM	569.83 ± 426.84	160.99 ± 92.63	<0.0001 *
F > 50 HpT vs. F ≤ 50 DCM	456.1 ± 471.90	202.15 ± 130.32	<0.0001 *
F > 50 HpT vs. F > 50 DCM	456.1 ± 471.90	160.99 ± 92.63	<0.0001 *
M ≤ 50 vs. M ≤ 50 DCM	699.24 ± 452.72	156.77 ± 91.17	<0.0001 *
M ≤ 50 vs. M > 50 DCM	699.24 ± 452.72	190.19 ± 128.23	<0.0001 *
M ≤ 50 HpT vs. M ≤ 50 DCM	712.73 ± 581.31	156.77 ± 91.17	<0.0001 *
M ≤ 50 HpT vs. M > 50 DCM	712.73 ± 581.31	190.19 ± 128.23	<0.0001 *
M > 50 vs. M ≤ 50 DCM	561.33 ± 354.90	156.77 ± 91.17	<0.0001 *
M > 50 vs. M > 50 DCM	561.33 ± 354.90	190.19 ± 128.23	<0.0001 *
M > 50 HpT vs. M ≤ 50 DCM	734.47 ± 497.22	156.77 ± 91.17	<0.0001 *
M > 50 HpT vs. M > 50 DCM	734.47 ± 497.22	190.19 ± 128.23	<0.0001 *
***MYH7***			
Pairwise comparisons	Mean ± SD		*p*-value
F ≤ 50 vs. F ≤ 50 DCM	2253.2 ± 981.41	3076.46 ± 1147.24	*0.018*
F ≤ 50 vs. F > 50 DCM	2253.2 ± 981.41	2613.48 ± 794.37	0.097
F > 50 vs. F ≤ 50 DCM	2281.46 ± 1171.00	3076.46 ± 1147.24	*0.023*
F > 50 vs. F > 50 DCM	2281.46 ± 1171.00	2613.48 ± 794.37	0.185
F > 50 HpT vs. F ≤ 50 DCM	2301.24 ± 872.76	3076.46 ± 1147.24	0.0004 *
F > 50 HpT vs. F > 50 DCM	2301.24 ± 872.76	2613.48 ± 794.37	0.058
M ≤ 50 vs. M ≤ 50 DCM	1535.29 ± 712.14	2736.27 ± 986.46	0.0001 *
M ≤ 50 vs. M > 50 DCM	1535.29 ± 712.14	2743.73 ± 890.50	<0.0001 *
M ≤ 50 HpT vs. M ≤ 50 DCM	2157.46 ± 650.84	2736.27 ± 986.46	*0.037*
M ≤ 50 HpT vs. M > 50 DCM	2157.46 ± 650.84	2743.73 ± 890.50	*0.012*
M > 50 vs. M ≤ 50 DCM	2590.00 ± 1458.18	2736.27 ± 986.46	0.365
M > 50 vs. M > 50 DCM	2590.00 ± 1458.18	2743.73 ± 890.50	0.290
M > 50 HpT vs. M ≤ 50 DCM	2142.65 ± 1032.92	2736.27 ± 986.46	0.0006 *
M > 50 HpT vs. M > 50 DCM	2142.65 ± 1032.92	2743.73 ± 890.50	0.001 *

Statistical analysis was performed using the Mann–Whitney U test; *p* ≤ 0.05 was considered statistically significant; *italicized p*-values indicate statistical significance at the 0.05 level, and asterisks (*) denote *p*-values that remained significant after Hochberg correction for multiple testing. Abbreviations: F ≤ 50—Females aged ≤ 50 years (including age 50 y); F > 50—Females aged > 50 years; F ≤ 50 DCM—Females aged ≤ 50 years with dilated cardiomyopathy; F > 50 DCM—Females aged > 50 years with dilated cardiomyopathy; F > 50 HpT—Females aged > 50 years with hypertension; M ≤ 50—Males aged ≤ 50 years (including age 50 y); M > 50—Males aged > 50 years; M ≤ 50 DCM—Males aged ≤ 50 years with dilated cardiomyopathy; M > 50 DCM—Males aged > 50 years with dilated cardiomyopathy; M ≤ 50 HpT—Males aged ≤ 50 years with hypertension; M > 50 HpT—Males aged > 50 years with hypertension.

**Table 6:** Pairwise comparisons of *MYH6* and *MYH7* gene expression between sexes: Control groups with different age and hypertension combinations compared to diseased groups of the opposite gender.

*MYH6*			
Pairwise Comparisons	Mean ± SD		*p*-Value
F ≤ 50 vs. M ≤ 50 DCM	881.3 ± 829.37	156.77 ± 91.17	<0.0001 *
M ≤ 50 vs. F ≤ 50 DCM	202.15 ± 130.32	699.24 ± 452.72	<0.0001 *
F ≤ 50 vs. M > 50 DCM	881.3 ± 829.37	190.19 ± 128.23	<0.0001 *
M ≤ 50 vs. F > 50 DCM	160.99 ± 92.63	699.24 ± 452.72	<0.0001 *
F > 50 vs. M ≤ 50 DCM	569.83 ± 426.84	156.77 ± 91.17	<0.0001 *
M > 50 vs. F ≤ 50 DCM	202.15 ± 130.32	561.33 ± 354.90	<0.0001 *
F > 50 HpT vs. M ≤ 50 DCM	456.1 ± 471.90	156.77 ± 91.17	<0.0001 *
M > 50 HpT vs. F ≤ 50 DCM	202.15 ± 130.32	734.47 ± 497.22	<0.0001 *
F > 50 HpT vs. M > 50 DCM	456.1 ± 471.90	190.19 ± 128.23	<0.0001 *
M > 50 HpT vs. F > 50 DCM	160.99 ± 92.63	734.47 ± 497.22	<0.0001 *
F > 50 vs. M > 50 DCM	569.83 ± 426.84	190.19 ± 128.23	<0.0001 *
M > 50 vs. F > 50 DCM	160.99 ± 92.63	561.33 ± 354.90	<0.0001 *
F ≤ 50 DCM vs. M ≤ 50 HpT	202.15 ± 130.32	712.73 ± 581.31	<0.0001 *
F > 50 DCM vs. M ≤ 50 HpT	160.99 ± 92.63	712.73 ± 581.31	<0.0001 *
***MYH7***			
Pairwise comparisons	Mean ± SD		*p*-value
F ≤ 50 vs. M ≤ 50 DCM	2253.2 ± 981.41	2736.27 ± 986.46	0.107
M ≤ 50 vs. F ≤ 50 DCM	1535.29 ± 712.14	3076.46 ± 1147.24	<0.0001 *
F ≤ 50 vs. M > 50 DCM	2253.2 ± 981.41	2743.73 ± 890.50	*0.034*
M ≤ 50 vs. F > 50 DCM	1535.29 ± 712.14	2613.48 ± 794.37	<0.0001 *
F > 50 vs. M ≤ 50 DCM	2281.46 ± 1171.00	2736.27 ± 986.46	0.073
M > 50 vs. F ≤ 50 DCM	2590.00 ± 1458.18	3076.46 ± 1147.24	0.134
F > 50 HpT vs. M ≤ 50 DCM	2301.24 ± 872.76	2736.27 ± 986.46	*0.029*
M > 50 HpT vs. F ≤ 50 DCM	2142.65 ± 1032.92	3076.46 ± 1147.24	0.0012 *
F > 50 HpT vs. M > 50 DCM	2301.24 ± 872.76	2743.73 ± 890.50	0.007 *
M > 50 HpT vs. F > 50 DCM	2142.65 ± 1032.92	2613.48 ± 794.37	*0.015*
F > 50 vs. M > 50 DCM	2281.46 ± 1171.00	2743.73 ± 890.50	0.065
M > 50 vs. F > 50 DCM	2613.48 ± 794.37	2 590.00 ± 1458.18	0.418
F ≤ 50 DCM vs. M ≤ 50 HpT	3076.46 ± 1147.24	2157.46 ± 650.84	0.003 *
F > 50 DCM vs. M ≤ 50 HpT	2613.48 ± 794.37	2157.46 ± 650.84	0.043

Statistical analysis was performed using the Mann–Whitney U test; *p* ≤ 0.05 was considered statistically significant; *italicized p*-values indicate statistical significance at the 0.05 level, and asterisks (*) denote *p*-values that remained significant after Hochberg correction for multiple testing. Abbreviations: F ≤ 50—Females aged ≤ 50 years (including age 50 y); F > 50—Females aged > 50 years; F ≤ 50 DCM—Females aged ≤ 50 years with dilated cardiomyopathy; F > 50 DCM—Females aged > 50 years with dilated cardiomyopathy; F > 50 HpT—Females aged > 50 years with hypertension; M ≤ 50—Males aged ≤ 50 years (including age 50 y); M > 50—Males aged > 50 years; M ≤ 50 DCM—Males aged ≤ 50 years with dilated cardiomyopathy; M > 50 DCM—Males aged > 50 years with dilated cardiomyopathy; M ≤ 50 HpT—Males aged ≤ 50 years with hypertension; M > 50 HpT—Males aged > 50 years with hypertension.

## Data Availability

The original contributions presented in this study are included in the article/Supplementary Material. Further inquiries can be directed to the corresponding author.

## Supplementary Materials

The following supporting information can be downloaded at: https://www.mdpi.com/article/10.3390/jcdd12110447/s1, Additional File S1: Separation into age groups, Additional File S2: Separation into age and hypertension groups, Additional File S3: Correlation analysis within nonfailing and DCM groups, Additional File S4: Thresholded groups.
